# Causal relationship between gut microbiota and polycystic ovary syndrome: a literature review and Mendelian randomization study

**DOI:** 10.3389/fendo.2024.1280983

**Published:** 2024-02-01

**Authors:** Junwei Sun, Mingyu Wang, Zhisheng Kan

**Affiliations:** ^1^ Department of Neurosurgery, National Cancer Center/National Clinical Research Center for Cancer/Cancer Hospital & Shenzhen Hospital, Chinese Academy of Medical Sciences and Peking Union Medical College, Shenzhen, China; ^2^ Department of Neurosurgery, Peking University China-Japan Friendship School of Clinical Medicine, Beijing, China; ^3^ Department of Radiology, National Cancer Center/National Clinical Research Center for Cancer/Cancer Hospital & Shenzhen Hospital, Chinese Academy of Medical Sciences and Peking Union Medical College, Shenzhen, China

**Keywords:** gut microbiota, polycystic ovarian syndrome, review, Mendelian randomization, causation

## Abstract

**Introduction:**

Numerous studies have suggested an association between gut microbiota and polycystic ovarian syndrome (PCOS). However, the causal relationship between these two factors remains unclear.

**Methods:**

A review of observational studies was conducted to compare changes in gut microbiota between PCOS patients and controls. The analysis focused on four levels of classification, namely, phylum, family, genus, and species/genus subgroups. To further investigate the causal relationship, Mendelian randomization (MR) was employed using genome-wide association study (GWAS) data on gut microbiota from the MiBioGen consortium, as well as GWAS data from a large meta-analysis of PCOS. Additionally, a reverse MR was performed, and the results were verified through sensitivity analyses.

**Results:**

The present review included 18 observational studies that met the inclusion and exclusion criteria. The abundance of 64 gut microbiota taxa significantly differed between PCOS patients and controls. Using the MR method, eight bacteria were identified as causally associated with PCOS. The protective effects of the genus *Sellimonas* on PCOS remained significant after applying Bonferroni correction. No significant heterogeneity or horizontal pleiotropy was found in the instrumental variables (IVs). Reverse MR analyses did not reveal a significant causal effect of PCOS on gut microbiota.

**Conclusion:**

The differences in gut microbiota between PCOS patients and controls vary across observational studies. However, MR analyses identified specific gut microbiota taxa that are causally related to PCOS. Future studies should investigate the gut microbiota that showed significant results in the MR analyses, as well as the underlying mechanisms of this causal relationship and its potential clinical significance.

## 1 Introduction

Polycystic ovary syndrome (PCOS) is a highly prevalent endocrine disorder in females, particularly among women of reproductive age. It is estimated that the incidence of PCOS in women of reproductive age ranges from 4% to 20% (1–3). Currently, Rotterdam criteria are recommended for the diagnosis of PCOS, which requires the presence of at least two of the following manifestations: oligo-anovulation, hyperandrogenism, and polycystic ovarian morphology (PCOM). However, the diagnosis of PCOS is challenging due to the diverse and varying features associated with this syndrome. The heterogeneity in the presentation of PCOS is observed in various aspects, including ultrasound assessment of ovarian morphology, overall length of the menstrual cycle, length of follicular phase, and length of luteal phase. Although the etiology of PCOS is not fully understood, some scholars have proposed possible mechanisms, including ones related to genetics, endocrinology, and metabolism (4–6). Tremellen et al. introduced the Dysbiosis of Gut Microbiota (DOGMA) theory in 2012, proposing that intestinal microorganisms may contribute to the typical manifestations of PCOS (7). Several observational studies have provided evidence supporting the association between PCOS and gut microbiota (8–10). For example, a significant reduction in the phylum *Tenericutes* has been reported in women with PCOS (11). PCOS patients have shown an increase in *Bacteroides vulgatus*, possibly influenced by a gut microbiota/bile acid/interleukin-22 axis (12). Furthermore, several studies have suggested that probiotic supplementation may be effective in treating PCOS, highlighting the potential therapeutic value of microbial treatment (13, 14). However, the findings of observational studies on the gut microbiota and PCOS are inconsistent, and it remains unclear whether there is a causal relationship between PCOS and the gut microbiota, as well as a reverse causal relationship between the two.

Investigating the causal relationship between gut microbiota and PCOS holds potential clinical value. It is important to conduct a comprehensive review of observational studies on the relationship between gut microbiota and PCOS, as well as to further explore the causal relationship between the two. Mendelian randomization (MR) is an analytical approach used to determine the causal association between an exposure or risk factor and a clinically relevant outcome, particularly when a randomized controlled trial is not feasible and observational studies may have biased associations due to confounding or reverse causality (15). MR is based on three assumptions and is considered a cost-effective and time-efficient approach with the use of publicly accessible genome-wide association study (GWAS) data (16, 17). MR analysis allows the determination of the causal relationship between gut microbiota and PCOS.

In the present study, a review of observational studies on changes in gut microbiota in patients with PCOS was conducted. Furthermore, two-sample MR and reverse MR analyses were performed to explore the bidirectional causal relationship between gut microbiota and PCOS.

## 2 Materials and methods

### 2.1 Literature search strategy and study selection criteria

Cohort and cross-sectional studies comparing changes in the gut microbiota of PCOS patients and controls were searched from inception to May 10, 2023. The following search terms were used: “gut microbiota,” “gastrointestinal microbiome,” “intestinal flora,” “microbiome,” and “microbiota” combined with “PCOS” and “polycystic ovary syndrome.” The inclusion criteria for the literature were as follows: (i) cohort studies or cross-sectional studies that compared gut microbiota changes between PCOS patients and healthy controls, with clear definitions of the subgroups in these studies; (ii) data included the first author, year of publication, country, study type, diagnostic criteria, sample size, age range, microbiota analysis technique, and statistical results related to the gut microbiota; (iii) fecal samples were used for gut microbiota analysis; and (iv) methods for identifying gut microbiota were clearly described. The exclusion criteria were as follows: (i) individual bacterial taxa not provided; (ii) any systematic analyses, reviews, case reports, or conference reports; and (iii) original data that could not be extracted from the literature.

### 2.2 Data extraction and quality assessment

Data were obtained from the texts, tables, and figures of each study. The following data were collected: first author, year of publication, country, study type, diagnostic criteria, sample size, age range, microbiota analysis technique, and statistical results related to the gut microbiota. All outcomes from the included studies were categorized into tables, offering a comprehensive overview of the variations in the gut microbiota between subjects with PCOS and control subjects. The tables provided detailed information from the phylum level down to the species level. Regarding the composition of the gut microbiota, a descriptive literature synthesis was performed, considering the variations in age, body mass index (BMI), assessment methods, small sample sizes, limited data, and the quality of the studies included. The risk of bias assessment tool for nonrandomized studies (RoBANS) was used to evaluate the methodological quality and risk of bias of each included study. The RoBANS tool contains six domains and is a valid tool for evaluating nonrandomized controlled trials (18).

### 2.3 MR study design and data sources

**Figure 1:**
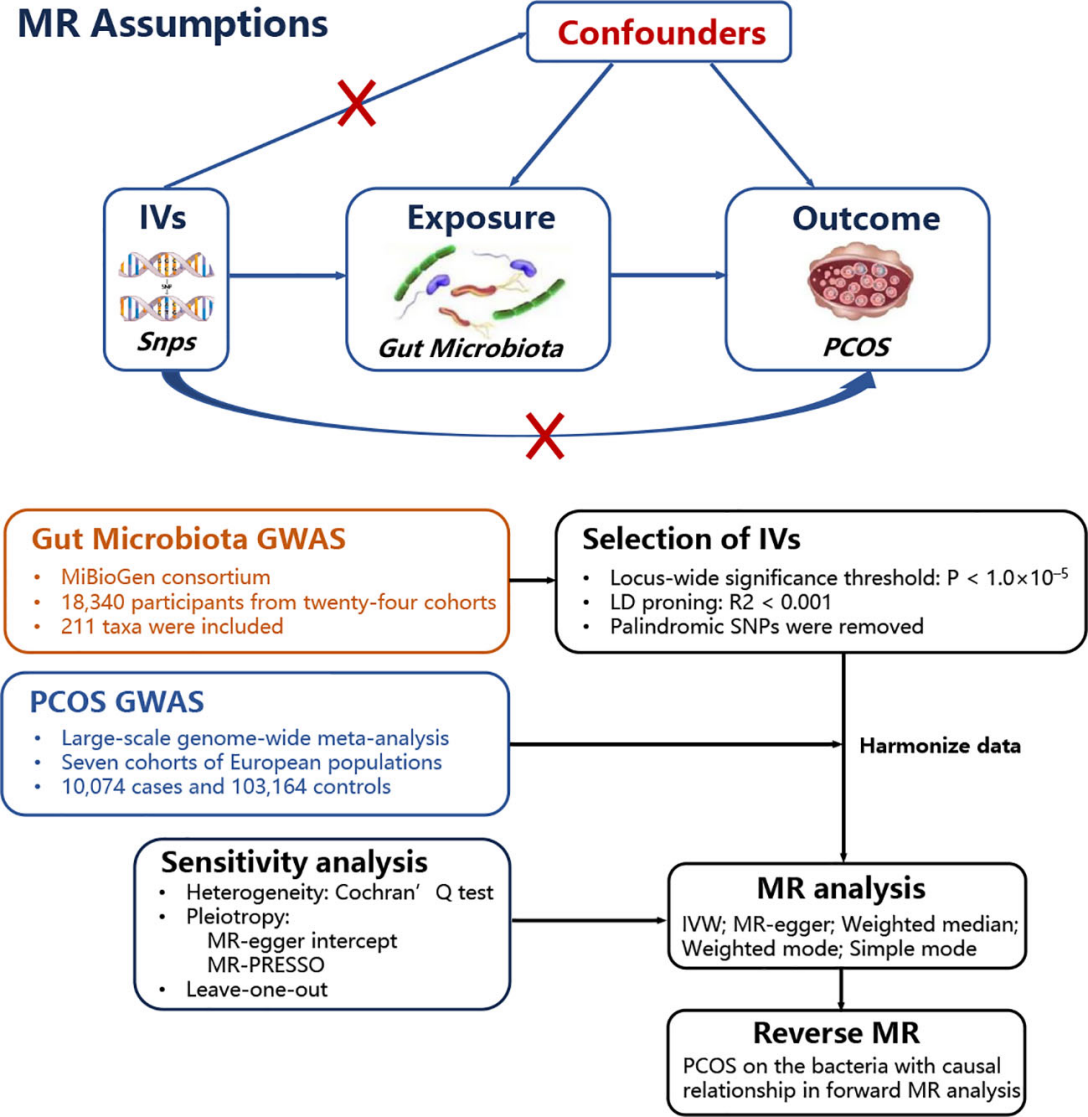
MR basic assumptions and overview of MR analyses process. The red X cross means that the IVs (instrumental variables) cannot influence the outcome throuth this path.

A two-sample MR study was performed to investigate the causal relationship between gut microbiota and PCOS. Genetic variants were used as instrumental variables (IVs) for the exposure because they are randomly allocated and not influenced by reverse causation or other confounding factors. The availability of publicly accessible GWAS data on gut microbiota and PCOS allowed efficient screening for suitable genetic IVs, making the two-sample MR approach cost-effective and time-efficient. A positive result from the MR analysis would provide support for a causal relationship between gut microbiota and PCOS. Additionally, a reverse MR was performed to examine whether PCOS also contributes to changes in gut microbiota. Valid estimates from the MR analyses were obtained when the following assumptions were satisfied: (i) a correlation between the single nucleotide polymorphisms (SNPs) and gut microbiota; (ii) SNPs influenced PCOS only through their impact on gut microbiota; and (iii) SNPs were not influenced by any confounding factors that may affect the relationship between gut microbiota and PCOS. **Figure 1** shows the MR analysis design that accompanied the basic MR assumptions.

The largest known GWAS dataset for gut microbiota was obtained from the MiBioGen consortium. The GWAS data involved 24 cohorts, consisting of 18,340 participants, most of whom (n = 13,266) were of European descent. The authors used microbiota quantitative trait loci (mbQTL) mapping analysis to identify host genetic variants related to the abundance of gut microbiota. The V1–V2, V3–V4, and V4 variable regions of the 16S rRNA gene were used to determine microbial composition. The GWAS data included 211 taxa from 9 phyla, 16 classes, 20 orders, 35 families, and 131 genera. Additional details about the microbiota data have been previously reported (19), and the comprehensive GWAS data is available at https://mibiogen.gcc.rug.nl/. GWAS data for PCOS were obtained from a large meta-analysis conducted by Day et al. (20), which involved 113,238 subjects, including 10,074 cases and 103,164 controls, all of whom were of European ancestry. Taking into account the characteristics of the GWAS of gut microbiota, MR analysis was performed at the five taxa levels (phylum, class, order, family, and genus) of gut microbiota. The GWAS data used in this study received ethical approval from their respective institutions and were publicly available online.

### 2.4 IV extraction and statistical analysis

The selection of IVs was based on the following criteria: (i) if P<5 × 10^−8^ was taken as the genome-wide significance threshold, the number of selected IV SNPs was too small; therefore, P<1.0 × 10^−5^ was taken as the threshold for screening IV in this study (21–24); (ii) based on the Europe-based 1,000 Genome project, a clustering distance of 10,000 kb and a threshold of r^2^<0.001 were used in linkage disequilibrium (LD) analysis; and (iii) palindromic SNPs were removed to avoid the influence of alleles.

In the present study, five high-efficiency MR analysis methods were used, and the inverse variance weighted (IVW) method was the main MR method. The IVW method is based on the three assumptions of MR and the idea of a randomized experiment, and it estimates the causality using genetic variation to explore the causality in observational data. The key advantage of the IVW method is its utilization of naturally occurring genetic variation as an IV to estimate the causal effect between exposure and outcomes. Genetic variation adheres to the principle of random distribution, allowing it to simulate a random experiment (25). However, it is important to note that the IVW method is not without limitations. In addition to the IVW method, four other MR analysis methods are commonly used as complementary methods, including the MR-Egger, weighted median, weighted mode, and simple mode methods. The specific principles, advantages, and disadvantages of each method have been previously reported (16, 26, 27).

Because two-sample MR has several limitations that can potentially impact the reliability of the MR results, effective testing methods were utilized to verify the results in the present study. Horizontal pleiotropy refers to when IVs have a direct impact on PCOS through alternative pathways, bypassing the influence of gut microbiota. MR-Pleiotropy RESidual Sum and Outlier (MR-PRESSO) was used to examine the outliers that may indicate pleiotropic biases. If the SNPs used as IVs had significant heterogeneity, the results of MR may be biased. The heterogeneity of the selected SNPs was assessed using Cochran’s Q statistic and leave-one-out sensitivity analysis. Weak IVs refer to the SNPs that are associated with exposure, but the strength of the association is not high. The strength of the IVs was examined using the F-statistic, which was calculated with the following formula $F=\;\frac{R^{2}\times \left(N-1-K\right)}{\left(1-R^{2}\right)\times K}$ : where *R^2^* is the fraction of variance in exposure explained by IVs; *K* is the number of IVs; and *N* is the sample size. An F-statistic greater than 10 indicates that the corresponding IV was not considered a weak IV (28). To obtain a more stringent interpretation of causality, Bonferroni correction based on 211 bacterial taxa was used as follows: 0.05/211 (3.81 × 10^−4^).

Reverse MR analysis was conducted on the bacteria identified as causally associated with PCOS in the forward MR analysis to examine the reverse causality between the gut microbiota and PCOS. The methods and settings used in reverse MR analysis were consistent with those used in the forward MR analysis.

R software (version 4.3.0) was used for statistical analysis.

## 3 Results

### 3.1 Literature search and basic characteristics

**Figure 2:**
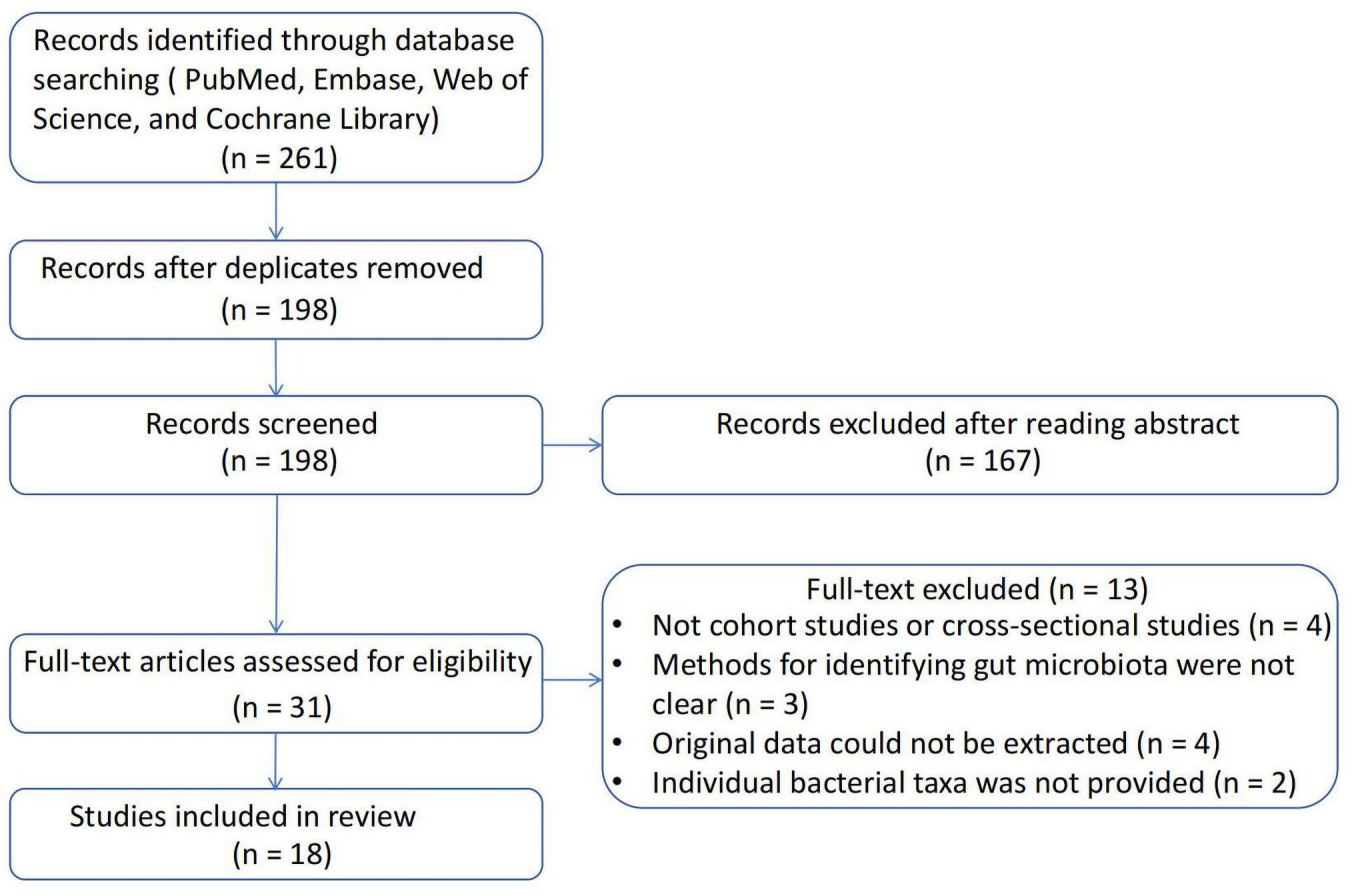
Flowchart of the literature selection.

A total of 261 studies were identified using the search strategy. After removing 63 duplicate studies, the remaining 198 studies were reviewed based on the inclusion and exclusion criteria. Ultimately, 18 articles were included in the present study. The process of literature screening is illustrated in **Figure 2**, and a summary of the included studies is shown in **Table 1**. Four studies were cross-sectional studies (12, 32, 33, 40), and 18 studies were cohort studies (11, 29–31, 34–39, 41–44). Most studies used the Rotterdam criteria for the diagnosis of PCOS, except for the study by Jobira, which used the National Institutes of Health (NIH) criteria (35). The study by Lüll et al. did not specify which diagnostic criteria were used (40). In addition, 16S rRNA gene sequencing with different region specifications was used as a microbiome assessment method in most studies, and whole-genome shotgun sequencing was used in the studies by Qi and Chu (12, 37). Seven studies divided participants into PCOS and healthy controls (11, 12, 30, 32, 33, 38, 40, 44), whereas the other studies subdivided participants into subgroups based on weight, insulin resistance, and other conditions. The studies were conducted in the following regions: 11 studies were conducted in East Asia (China) (12, 29, 31, 33, 34, 36, 37, 39, 41, 42, 44); four studies were conducted in Europe (11, 30, 32, 40); one study was conducted in North America (America) (35); and one study was conducted in Western Asia (Turkey) (38).

**Table 1:** Characteristics of the observational studies included in the review.

First Author	Year	Country	Study Design	Definition of PCOS	Major Microbiome Identification Method	Group	Patients, n	Age, years
**Liu** (29)	2017	China	Cross-sectional study	Rotterdam criteria	16S rRNA gene sequencing	Obese PCOS	21	29.3 ± 6.5
						Non-obese PCOS	12	25.5 ± 4.3
						Obese control	6	33 ± 5.4
						Non-obese control	9	32.2 ± 5.9
**Lindheim** (11)	2017	Austria	Cross-sectional study	Rotterdam criteria	16S rRNA gene sequencing	PCOS	24	27 (5.9)
						Healthy control	20	32 (12.0)
**Insenser** (30)	2018	Spain	Cross-sectional study	Rotterdam criteria	16S rRNA gene sequencing	PCOS	15	26.7 ± 7.2
						Healthy control	16	27.3 ± 5.2
**Zeng** (31)	2018	China	Cross-sectional study	Rotterdam criteria	16S rRNA gene sequencing	PCOS with insulin resistance	9	25.1 ± 4.3
						PCOS without insulin resistance	8	26.1 ± 7.1
						Healthy control	8	26.4 ± 3.9
**Torres** (32)	2018	Poland	Cohort study	Rotterdam criteria	16S rRNA gene sequencing	PCOS	73	27.4 ± 4.9
						Healthy control	48	29.4 ± 4.9
						PCOM Only	42	29.8 ± 5.3
**Qi** (12)	2019	China	Cohort study	Rotterdam criteria	Whole-genome shotgun sequencing	PCOS	50	20-40
						Healthy control	43	20-40
**Zhang** (33)	2019	China	Cohort study	Rotterdam criteria	16S rRNA gene sequencing	PCOS	38	27.6 ± 3.8
						Healthy control	26	26.7 ± 2.0
**Zhou** (34)	2020	China	Cross-sectional study	Rotterdam criteria	16S rRNA gene sequencing	Obese PCOS	30	26.9 ± 4.9
						Non-obese PCOS	30	25.1 ± 4.3
						Obese control	11	25.3 ± 1.6
						Non-obese control	30	22.1 ± 1.6
**Jobira** (35)	2020	America	Cross-sectional study	NIH	16S rRNA gene sequencing	Obese PCOS	37	16.1 ± 0.3
						Obese control	21	14.5 ± 0.4
**Liang** (36)	2020	China	Cross-sectional study	Rotterdam criteria	16S rRNA gene sequencing	Obese PCOS	8	27.1 ± 3.5
						Non-obese PCOS	10	25.7 ± 3.5
						Healthy control	9	27.9 ± 3.6
**Chu** (37)	2020	China	Cross-sectional study	Rotterdam criteria	Whole-genome shotgun sequencing	Non-overweight PCOS	7	27.14 ± 4.56
						Non-overweight control group	7	30.29 ± 3.90
						Overweight PCOS	7	29.14 ± 2.87
						Overweight control group	7	28.57 ± 2.72
**Eyupoglu** (38)	2020	Turkey	Cross-sectional study	Rotterdam criteria	16S rRNA gene sequencing	PCOS	17	20 (19–22)
						Healthy control	15	22 (18–27)
**Chen** (39)	2021	China	Cross-sectional study	Rotterdam criteria	16S rRNA gene sequencing	PCOS-LB	98	29.48 ± 3.39
						PCOS-HB	50	29.64 ± 4.06
						Healthy control	38	29.26 ± 4.18
**Lüll** (40)	2021	Estonia	Cohort study	–	16S rRNA gene sequencing	PCOS	102	46
						Healthy control	201	46
**Liang** (41)	2021	China	Cross-sectional study	Rotterdam criteria	16S rRNA gene sequencing	Lean PCOS	10	24.13 ± 2.45
						Overweight PCOS	10	28.94 ± 6.13
						Lean healthy control	10	25.08 ± 3.59
						Overweight healthy control	10	30.12 ± 5.20
**Dong** (42)	2021	China	Cross-sectional study	Rotterdam criteria	16S rRNA gene sequencing	PCOS	37	26-35
						Healthy control	45	28-35
**Mammadova** (43)	2021	Turkey	Cross-sectional study	Rotterdam criteria	16S rRNA gene sequencing	Lean PCOS	24	19.0-22.5
						Lean healthy control	22	22.0-24.25
**Li** (44)	2022	China	Cross-sectional study	Rotterdam criteria	16S rRNA gene sequencing	PCOS	31	24.35 ± 4.48
						Healthy control	27	27.0 ± 4.90

NIH (National Institutes of Health); PCOS (polycystic ovarian syndrome); PCOM (polycystic ovarian morphology); PCOS-LB (normal body mass index (BMI) PCOS); PCOS-HB (high body mass index (BMI) PCOS).

Age, years, mean ± standard deviation, median (range), or age lower limit–age upper limit.

“-“ indicates that the literature did not provide this information.

### 3.2 Summary of bacterial taxa changes in observational studies

All studies included in the review utilized high-throughput sequencing methods, specifically whole-genome shotgun sequencing and 16S rRNA gene sequencing. The abundance of gut microbiota was expressed using various parameters, including observed operating taxonomic unit (OTU) counts, richness, Chaos1, Shannon index, and evenness. To determine changes in the abundance of specific gut microbiota taxa, a species was considered to be increased or decreased if its relative abundance differed significantly (P< 0.05) at the phylum, family, genus, or species/genus subgroups levels.

None of the included studies reported differential gut microbiota taxa between PCOS patients and controls at the class and order levels. The groups for comparison were those with PCOS and those without PCOS, with or without involved subgroups. Three studies reported a significant increase in the abundance of the following phyla: *Proteobacteria*, *Actinobacteria*, and *Fusobacteria* in PCOS patients (35, 39, 43). Moreover, the following phyla were decreased in PCOS patients in three studies: *Bacteroidetes*, *Firmicutes*, *Tenericutes*, *Proteobacteria*, and *Actinobacteria* (11, 35, 39). At the family taxonomic level, *Lactobacillaceae*, *Streptococcaceae*, *Erysipelotrichaceae*, and *Enterobacteriaceae* were significantly elevated in patients with PCOS in three studies (35, 43, 44), while *S24-7*, *Prevotellaceae*, *Porphyromonadaceae*, *Barnesiellaceae*, *Christensenellaceae*, *Aerococcaceae*, and *Pasteurellaceae* were significantly less abundant in five studies (11, 31, 35, 42, 44). Changes in the abundance of some families of gut microbiota in PCOS patients, including *Bacteroidaceae*, *Ruminococcaceae*, and *Lachnospiraceae*, were inconsistent in different studies (29, 31, 35, 36, 38, 44). Many significant differences were found at the genus level. The genus *Bacteroides* differed in abundance between PCOS and controls in six studies, with opposite results (12, 29, 33, 35, 37, 41). Moreover, the following genera were significantly increased in the PCOS group: *Ruminococcaceae UCG-002, Subdoligranulum, Lactobacillus*, *Oscillibacter, Catenibacterium, Kandleria, Coprococcus*, *Lactococcus*, *Megamonas, Eubacterium, Escherichia, Klebsiella*, and *Bifidobacterium* (29, 30, 33, 34, 36, 40, 41, 44). The following genera were significantly reduced in the PCOS group: *Prevotella, Akkermansia, Faecalibacterium, Ruminococcus, Blautia, Roseburia, Lachnospira, Fusicatenibacter, Erysipelatoclostridium, Abiotrophi*, and *Haemophilus* (29, 33, 37, 39, 42–44). Three genera, namely, *Parabacteroides, Clostridium*, and *Bifidobacterium*, showed opposite abundance changes in different studies (33, 35, 37, 40).

In addition, four studies examined changes in abundance at the species or genus subgroup taxa level. Among them, genus *Bacteroides coprophilus*, genus *Bacteroides fragilis*, genus *Ruminococcus gnavus*, species *Blautia* spp., and genus *Collinsella aerofaciens* had significant increases in PCOS patients (32, 33, 42). Moreover, the significantly reduced genera or species taxa included species *Odoribacter* spp., genus *Ruminococcus bromii.*, genus *Clostridium cluster XVII*, genus *Clostridium sensu stricto*, species *Anaerococcus* spp., and species *Bifidobacterium* spp. (29. 30, 43). **Table 2** summarizes the gut microbiota taxa at the four levels with significant differences among the included studies.

**Table 2:** Summary of representative taxa in PCOS patients compared to healthy controls in the included observational studies.

Phylum	Increasse	Decrease	Family	Increasse	Decrease	Genus	Increasee	Decrease	Species or Genus Subgroups subgsfdgdsssdffssubSubgroup	Increasse	Decrease
***Bacteroidetes***		Jobira2020	*Bacteroidaceae*	zeng 2018	Jobira2020	*Bacteroides*	Liu 2017	Jobira 2020	*Bacteroides coprophilus*	Torres 2018	
		Chen 2021					Qi 2019	Chu 2020	*Bacteroides fragilis*	Dong 2021	
							Zhang 2019		*Dialister succinatiphilus*	Dong 2021	
							Liang 2021				
			*S24-7*		Lindheim 2017						
			*Prevotellaceae*		Zeng 2018	*Prevotella*		Chu 2020	*Prevotella stercorea*	Dong 2021	
					Li 2022			Chen 2021			
			*Porphyromonadaceae*		Jobira2020	*Porphyromonas*			*Porphyromonas* spp.	Torres 2018	
			*Odoribacteraceae*			*Odoribacter*			*Odoribacter* spp.		Torres 2018
			*Tannerellaceae*			*Parabacteroides*	Zhang 2019	Jobira 2020			
			*Barnesiellaceae*		Dong 2021						
***Verrucomicrobia***			*Akkermansiaceae*			*Akkermansia*		Liu 2017			
***Firmicutes***		Chen 2021	*Ruminococcaceae*	Zeng 2018	Liu2017	*Faecalibacterium*		Zhang 2019	*Faecalibacterium prausnitzii*	Torres 2018	Zhang 2019
				Eyupoglu 2020	Li 2022	*Ruminococcus*		Liu 2017	*Ruminococcus bromii.*		Torres 2018
									*Ruminococcus gnavus*	Dong 2021	
						*RuminococcaceaeUCG002*	Lüll 2021				
						*Subdoligranulum*	Liang 2020				
			*Lactobacillaceae*	Jobira 2020		*Lactobacillus*	Zhang 2019				
			*Oscillospiraceae*			*Oscillibacter*	Zhang 2019				
			*Clostridiaceae*			*Clostridium*	Zhang 2019	Chu 2020	*Clostridium cluster XVII*		Mammadova 2021
									*Clostridium sensu stricto*		Mammadova 2021
			*Erysipelotrichaceae*			*Catenibacterium*	Insenser 2018				
						*Kandleria*	Insenser 2018				
			*Lachnospiraceae*	Zeng 2018	Liang2020	*Blautia*		Zhang 2019	*Blautia* spp.	Torres 2018	
						*Roseburia*		Mammadova 2021	*Roseburia* spp.	Dong 2021	Torres 2018
						*Lachnospira*		Zhang 2019			
						*Coprococcus*	Zhou 2020				
			*Peptoniphilaceae*			*Anaerococcus*			*Anaerococcus* spp.		Torres 2018
			*Streptococcaceae*	Jobira 2020		*Lactococcus*	Zhou 2020				
			*Selenomonadaceae*			*Megamonas*	Liang 2020				
			*Eubacteriaceae*			*Eubacterium*	Lüll 2021				
			*Christensenellaceae*		Dong 2021						
						*Fusicatenibacter*		Dong 2021			
			*Erysipelotrichaceae*	Mammadova 2021		*Erysipelatoclostridium*		Li 2022			
			*Aerococcaceae*		Li 2022	*Abiotrophia*		Li 2022			
			*Peptostreptococcaceae*								
***Tenericutes***		Lindheim 2017									
***Proteobacteria***	Mammadova 2021 d2021202122021 2021	Chen 2021	*Enterobacteriaceae*	Mammadova 2021		*Escherichia*	Zhang 2019				
				Li 2022			Liu 2017				
							Liang 2021				
						*Klebsiella*	Li 2022				
			*Pasteurellaceae*		Dong 2021	*Haemophilus*		Dong 2021			
***Actinobacteria***	Jobira 2020	Chen 2021	*Bifidobacteriaceae*			*Bifidobacterium*	Lüll 2021	Zhang 2019	Bifidobacterium spp.		Zhang 2019
			*Coriobacteriaceae*			*Collinsella*			Collinsella aerofaciens	Zhang 2019	
***Verrucomicrobia***			*Akkermansiaceae*			*Akkermansia*		Liu 2017			
***Fusobacteria***	Chen 2021										

The dietary patterns of populations from different regions vary, which can lead to differences in the species and abundance of gut microbiota. To compare the changes in gut microbiota of PCOS populations in different regions, the abundances of gut microbiota in populations from various countries were summarized (**Supplementary Table 1**).

### 3.3 Risk of bias assessment

The quality of the included studies was assessed using the RoBANS tool and is summarized in **Supplementary Table 2**. The included studies did not show a risk of exposure measurement bias, and they reported complete outcome data. Six studies had a high risk of bias in the selection of participants, and three studies involved confounding variables. In addition, 14 studies did not assess the risk of outcome blinding.

### 3.4 MR analysis results

In total, 2832 SNPs were identified as gut microbiota IVs. All SNPs had F-statistics greater than 10 (F ranged between 11.43 and 98.47), which indicated that there were no weak IVs. Additional information on the selected IVs is provided in **Supplementary Table 3**. In the forward MR Analysis, eight gut microbiota taxa were identified as having a significant causal relationship with PCOS using the IVM method, including one gut microbiota taxon at the family level and seven gut microbiota taxa at the genus level. Among them, four taxa had protective effects on PCOS, and four taxa were risk factors for PCOS.

**Figure 3:**
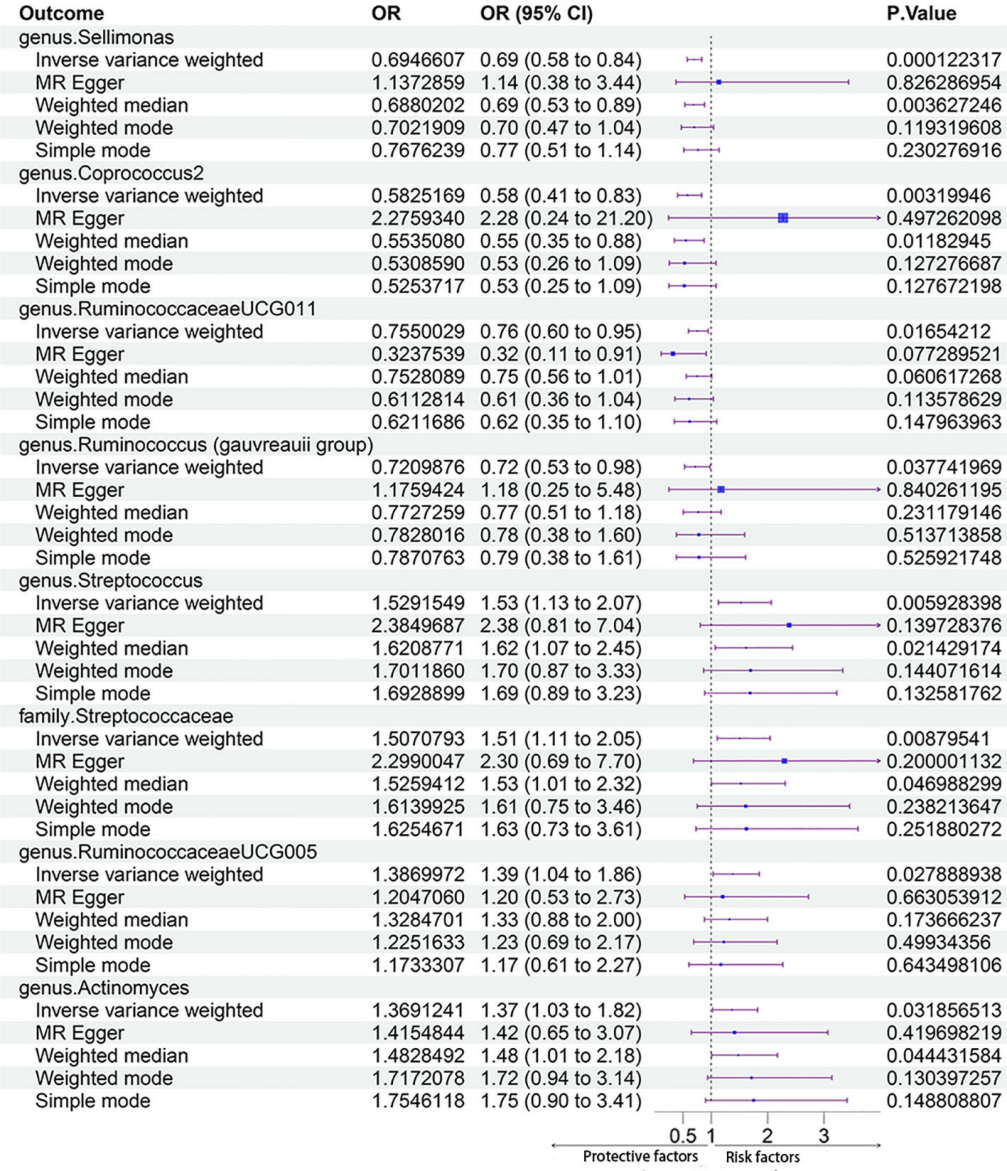
The results of MR analysis on the causal effect of the eight gut microbiota taxa on PCOS.

The MR estimates with the IVW method showed that some genera, including *Sellimonas* [odds ratio (OR) 0.69, 95% confidence interval (CI) 0.58 - 0.84, P = 1.22 × 10^-4^], *Coprococcus2* (OR 0.58, 95% CI 0.41 - 0.83, P = 3.20 × 10^-3^), *Ruminococcaceae UCG011* (OR 0.75, 95% CI 0.60 - 0.95, P = 1.62 × 10^-2^), and *Ruminococcus (Gauvreauii group)* (OR 0.72, 95% CI 0.53 - 0.98, P = 3.78 × 10^-2^), had protective effects on PCOS. IVM analysis showed that the risk gut microbiota taxa for PCOS were genus *Streptococcus* (OR 1.53, 95% CI 1.13 - 2.07, P = 5.93 × 10^-3^), family *Streptococcaceae* (OR 1.51, 95% CI 1.11 - 2.05, P = 8.76 × 10^-3^), genus *RuminococcaceaeUCG005* (OR 1.39, 95% CI 1.04 - 1.86, P = 2.79 × 10^-2^), and genus *Actinomyces* (OR, 1.37; 95% CI, 1.03 - 1.82; P = 3.19 × 10^-2^). After Bonferroni correction, genus *Sellimonas* remained significant in the MR analysis results (adjusted P = 2.57 × 10^-2^). **Figure 3** shows the MR analysis results of the causal effects of the eight gut microbiota taxa on PCOS. The results of the MR analysis on the causal relationship between the 211 gut microbiota taxa and PCOS are shown in **Supplementary Table 4**.

**Figure 4:**
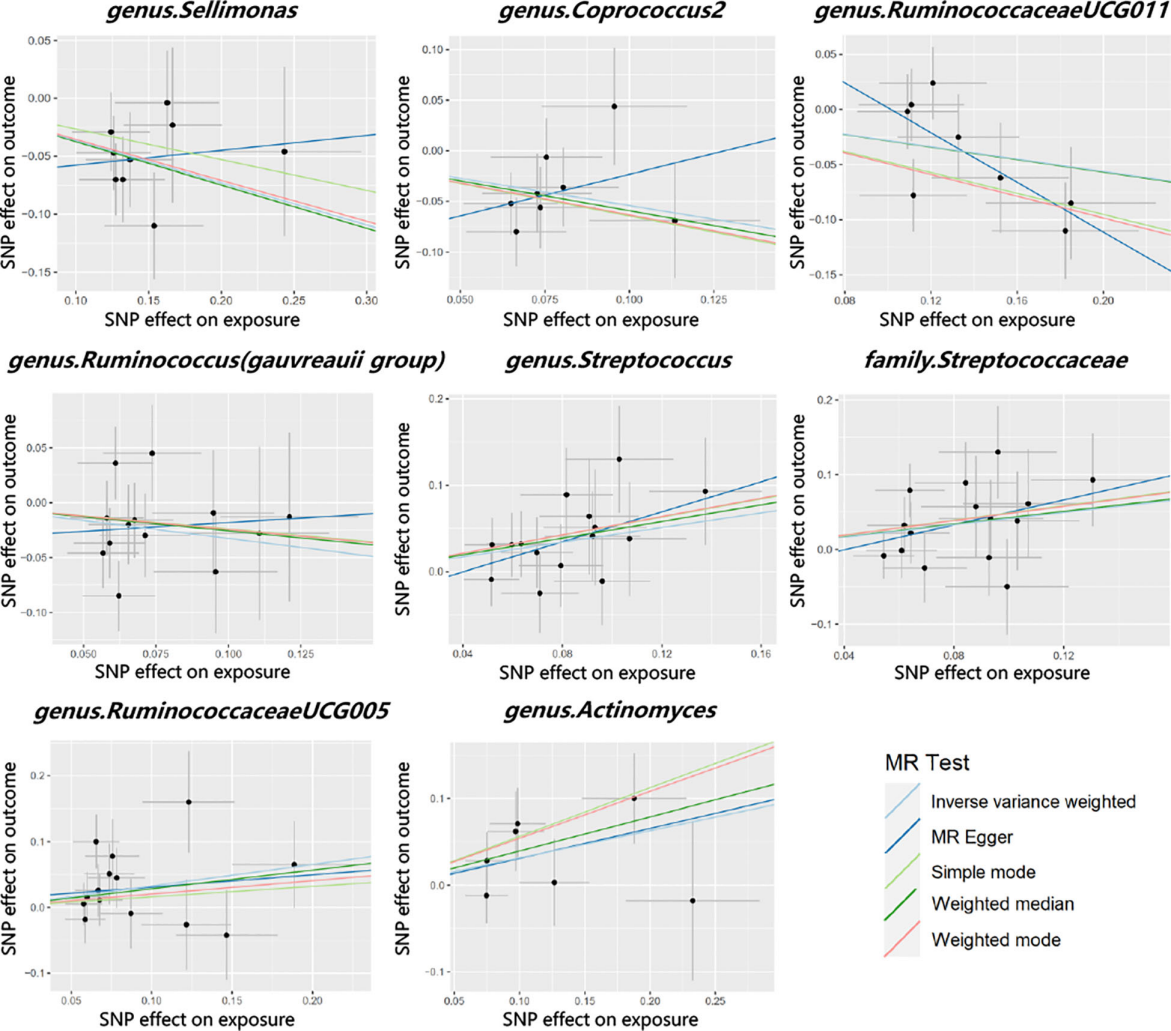
Scatter plots for the causal association between gut microbiota and PCOS.

**Figure 5:**
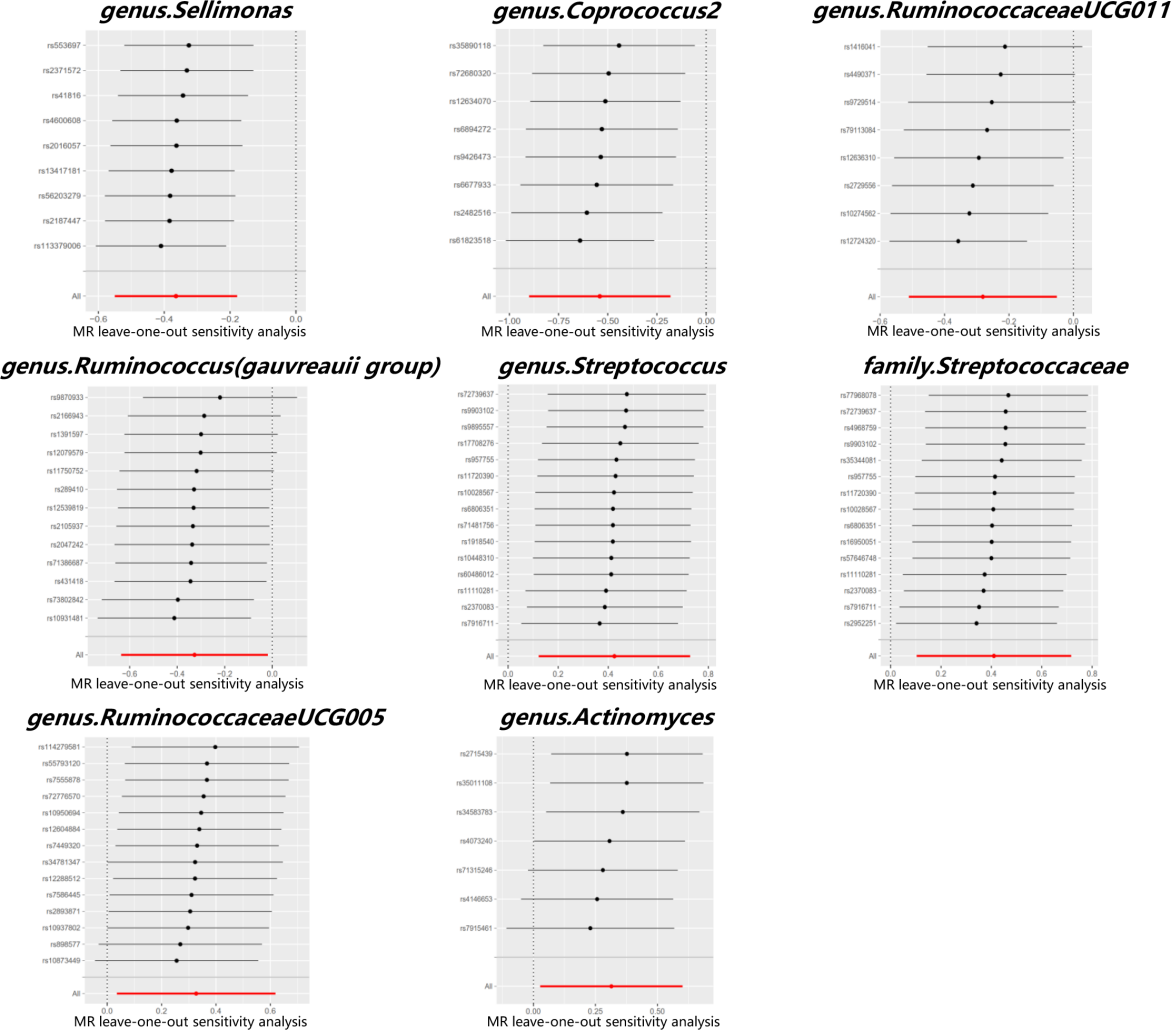
Leave-one-out plots for the causal association between gut microbiota and PCOS.

Cochran’s Q test did not identify significant heterogeneity in the IVs in the forward MR Analysis (**Supplementary Table 5**). Visual inspection of the scatter plots and leave-one-out plots of the IVs revealed no potential outliers for the gut microbiota taxa (**Figures 4**, **5**). Additionally, no horizontal pleiotropy was observed in the MR-Egger regression intercept analysis (**Supplementary Table 6**), and no significant outliers were found in the MR-PRESSO analysis (global test, P > 0.05) (**Supplementary Table 6**). Therefore, there was insufficient evidence for horizontal pleiotropy in the causal relationship between the eight gut microbiota taxa and PCOS.

In the reverse MR analysis, a significant causal relationship was not observed between PCOS and any of the eight gut microbiota taxa (**Supplementary Tables 7**, **8**). Cochran’s Q test revealed no significant heterogeneity in the IVs of patients with PCOS (**Supplementary Table 9**). In the reverse MR analysis, MR-Egger regression did not show significant results for horizontal pleiotropy (**Supplementary Table 10**).

## 4 Discussion

The human gut contains trillions of microbes, which have been recognized as significant environmental factors impacting human physiology and pathology (45). Following the discovery by Poretsky et al. that gut microbiota disorders are closely associated with metabolic abnormalities in PCOS patients, numerous studies have been conducted to investigate the connection between gut microbiota and PCOS (46–49). Regardless of the level of gut microbiota taxa, various observational studies have arrived at different and even opposite findings, as shown in **Table 2**. The significant variations in results can be attributed to the diversity among different observational studies, including differences in sample sizes, grouping criteria, and microbiological identification methods. It is important to consider that populations from different regions have varying dietary patterns, which can contribute to the inconsistent findings (50). Future research should prioritize the development of more effective methods to compare the gut microbiota composition among individuals from different geographical locations.

Observational studies make it challenging to determine whether these changes are a cause or consequence of PCOS. However, the utilization of MR and publicly available GWAS data has facilitated the identification of causal relationships between gut microbiota and PCOS (51, 52). In the present study, the IVM analysis results for the genus *Sellimonas* remained significant after applying the Bonferroni correction. Genus *Sellimonas* belongs to the family *Lachnospiraceae*, which is part of the phylum *Firmicutes.* Genus *Sellimonas* is an anaerobic gram-positive microbe that has received relatively little attention in previous research (53). Muñoz et al. found that the genus *Sellimonas* may be a marker of intestinal homeostasis, and its abundance gradually increases with the restoration of intestinal barrier function (54, 55). Genus *Coprococcus*, an important member of the phylum *Firmicutes*, is known for its production of butyric acid (56). Zhou et al. proposed that genus *Coprococcus2* is a distinguishing gut microbiota in obese PCOS patients (34). However, the present IVM analysis did not support this observation, which may be attributed to the distinct group specificity of gut microbiota (57). The genus *Ruminococcus*, belonging to the family *Ruminococcaceae*, has a potential role in the protective effect against PCOS. One possible explanation for this effect is that an increase in the genus *Ruminococcus* (*Gauvreauii group*) is strongly associated with improved insulin sensitivity in obese patients (58). Interestingly, genera *Streptococcus* and *Actinomyces* are significantly increased in the saliva and vagina, but not in the intestine, of PCOS patients (59, 60). The present MR study suggested that the observed relationship between gut microbiota and PCOS may not necessarily be causal. One possible explanation is that the gut microbiota, which has a causal relationship with PCOS, may not be detected due to its low abundance, as well as limitations in current detection and statistical methods. Nevertheless, the findings obtained through the MR method are noteworthy and warrant further investigation.

The gut microbiota/bile acid/interleukin-22 axis has been investigated as one of the possible potential mechanisms for this causal relationship (12, 61). Transplantation of fecal microbiota from women with PCOS or *B. vulgatus*-colonized recipient mice results in various negative effects on ovarian functions, insulin resistance, bile acid metabolism, interleukin-22 secretion, and fertility. IL-22 improves the PCOS phenotype. Some gut microbiota taxa are capable of inducing oxidative stress and an inflammatory response from mononuclear cells of women with PCOS by influencing the dietary trigger (62, 63). Oxidative stress and inflammation also damage the structure and function of intestinal permeability, leading to an increase in gram-negative bacteria in the blood. This activation of the immune system hampers the function of insulin receptors, which results in elevated insulin levels, subsequently leading to high levels of androgens and abnormal follicular development (64). Energy absorption may be a mechanism of gut microbiota leading to PCOS (65). Studies have indicated that more than half of PCOS patients are overweight or obese (11). Researchers have confirmed that the richness and phylogenetic diversity of gut microbiota in patients with PCOS are significantly reduced, and multiple meta-analyses have confirmed that this reduction is closely related to the obesity phenotype of PCOS (66, 67) Other possible mechanisms include the lipopolysaccharide metabolic pathway, short-chain fatty acid (SCFA) metabolic pathway, and choline pathway (68–70).

The present study of the relationship between gut microbiota and PCOS holds potential clinical significance. Firstly, changes in gut microbiota may serve as an indicator for the occurrence of PCOS, suggesting that gut microbiota may potentially be used for diagnostic purposes in PCOS. A functional predictive analysis conducted by Zhou et al. revealed significant differences in fecal microbes among obese PCOS patients, non-obese PCOS patients, and healthy individuals (34), highlighting significant differences in the gut microbiota and its predicted functions in obese and non-obese women with PCOS. The positive bacteria identified in the present MR study have promising potential as diagnostic bacteria for the occurrence and post-treatment effects of PCOS in the future. Moreover, alterations in gut microbiota have been found to be closely associated with various PCOS-related clinical parameters, including metabolic factors, sex hormones, and mediators of the brain-gut axis (29). Secondly, treatment of PCOS based on gut microbiota has attracted increasing attention. Animal experiments involving fecal microbiota transplantation (FMT) have demonstrated significant improvements in PCOS-related symptoms after PCOS mice receive feces from healthy mice. The use of FMT as a potential approach to treat PCOS holds promise for the future. Additionally, microbial agents, such as probiotics, prebiotics, and synbiotics, have shown potential therapeutic value in PCOS treatment (71, 72). The protective bacteria identified in the present MR study should be considered as potential sources of microbial agents in future studies. Moreover, studies have indicated that specific drugs used for PCOS treatment may achieve their intended effects by influencing changes in gut microbiota. These drugs include metformin, thiazolidinediones, and lipid-lowering drugs (73–75). Thus, the mediating role played by gut microbiota deserves attention.

## 5 Limitations

In reviewing observational studies, a major challenge is the difficulty in adjusting for potential confounding factors and other variables due to the significant heterogeneity among the studies. The complexity of the included population groups limited our ability to conduct a more rigorous quantitative systematic review of these studies. Additionally, the various gut microbiota patterns influenced by diet habits in different regions made it challenging to interpret the summarized results using MR analysis. Moreover, the GWAS data used in the present MR analysis was obtained from 24 cohorts in different countries. Due to the polymorphism observed among different human populations, the outcomes of GWAS may vary across these populations (76). Furthermore, the presence of genetic heterogeneity within each population can also impact the reliability of GWAS results. This limitation may affect the interpretation of the GWAS findings. In MR Studies, it is challenging to completely avoid LD. In addition, when using genes as IVs, biases may arise due to weak instruments, population stratification, and developmental compensation (77). Bonferroni correction was used as a multiple test in the present MR analysis. One advantage of this correction method is its effectiveness in controlling type I errors, but it may be overly conservative, which can increase type II error rates (78). Additionally, the strictness of Bonferroni may filter out potentially meaningful results.

## Data availability statement

The datasets presented in this study can be found in online repositories. The names of the repository/repositories and accession number(s) can be found in the article/**Supplementary Material**.

## Ethics statement

Ethical approval was not required for the study involving humans in accordance with the local legislation and institutional requirements. Written informed consent to participate in this study was not required from the participants or the participants’ legal guardians/next of kin in accordance with the national legislation and the institutional requirements.

## Author contributions

JS: Data curation, Formal analysis, Writing – original draft. MW: Methodology, Resources, Software, Writing – original draft. ZK: Supervision, Writing – review & editing.

## References

[1] The Rotterdam ESHRE/ASRM-sponsored PCOS consensus workshop group. Revised 2003 consensus on diagnostic criteria and long-term health risks related to polycystic ovary syndrome (PCOS). Hum Reprod (2004) 19:41–7. doi: 10.1093/humrep/deh098 14688154

[2] Escobar-Morreale HF Luque-Ramírez M González F. Circulating inflammatory markers in polycystic ovary syndrome: a systematic review and metaanalysis. Fertil Steril (2011) 95:1048–1058.e2. doi: 10.1016/j.fertnstert.2010.11.036 21168133 PMC3079565

[3] Dunaif A Segal KR Futterweit W Dobrjansky A. Profound peripheral insulin resistance, independent of obesity, in polycystic ovary syndrome. Diabetes (1989) 38:1165–74. doi: 10.2337/diab.38.9.1165 2670645

[4] Meier RK. Polycystic ovary syndrome. Nurs Clinics North America (2018) 53:407–20. doi: 10.1016/j.cnur.2018.04.008 30100006

[5] Hughes C Elgasim M Layfield R Atiomo W. Genomic and post-genomic approaches to polycystic ovary syndrome–progress so far: Mini Review. Hum Reprod (2006) 21:2766–75. doi: 10.1093/humrep/del222 16936300

[6] Goodarzi MO Dumesic DA Chazenbalk G Azziz R. Polycystic ovary syndrome: etiology, pathogenesis and diagnosis. Nat Rev Endocrinol (2011) 7:219–31. doi: 10.1038/nrendo.2010.217 21263450

[7] Tremellen K Pearce K. Dysbiosis of Gut Microbiota (DOGMA) – A novel theory for the development of Polycystic Ovarian Syndrome. Med Hypotheses (2012) 79:104–12. doi: 10.1016/j.mehy.2012.04.016 22543078

[8] Chavarro JE Rich-Edwards JW Rosner B Willett WC. A prospective study of dairy foods intake and anovulatory infertility. Hum Reprod (2007) 22:1340–7. doi: 10.1093/humrep/dem019 17329264

[9] Yadav H Jain S Sinha PR. Oral administration of dahi containing probiotic Lactobacillus acidophilus and Lactobacillus casei delayed the progression of streptozotocin-induced diabetes in rats. J Dairy Res (2008) 75:189–95. doi: 10.1017/s0022029908003129 18474136

[10] Luoto R Laitinen K Nermes M Isolauri E. Impact of maternal probiotic-supplemented dietary counselling on pregnancy outcome and prenatal and postnatal growth: a double-blind, placebo-controlled study. Br J Nutr (2010) 103:1792–9. doi: 10.1017/s0007114509993898 20128938

[11] Lindheim L Bashir M Münzker J Trummer C Zachhuber V Leber B. Alterations in gut microbiome composition and barrier function are associated with reproductive and metabolic defects in women with polycystic ovary syndrome (PCOS): A pilot study. PloS One (2017) 12:e0168390. doi: 10.1371/journal.pone.0168390 28045919 PMC5207627

[12] Qi X Yun C Sun L Xia J Wu Q Wang Y. Gut microbiota-bile acid-interleukin-22 axis orchestrates polycystic ovary syndrome. Nat Med (2019) 25:1225–33. doi: 10.1038/s41591-019-0509-0 PMC7376369 31332392

[13] Askari G Shoaei T Tehrani H Heidari-Beni M feizi A Esmaillzadeh A. Effects of probiotic supplementation on pancreatic β-cell function and c-reactive protein in women with polycystic ovary syndrome: A randomized double-blind placebo-controlled clinical trial. Int J Prev Med (2015) 6:27. doi: 10.4103/2008-7802.153866 25949777 PMC4387688

[14] Jamilian M Mansury S Bahmani F Heidar Z Amirani E Asemi Z. The effects of probiotic and selenium co-supplementation on parameters of mental health, hormonal profiles, and biomarkers of inflammation and oxidative stress in women with polycystic ovary syndrome. J Ovarian Res (2018) 11(1):80. doi: 10.1186/s13048-018-0457-1 30217229 PMC6137747

[15] Greenland S. An introduction to instrumental variables for epidemiologists. Int J Epidemiol (2017) 47:358–8. doi: 10.1093/ije/dyx275 29294084

[16] Carter AR Sanderson E Hammerton G Richmond RC Davey Smith G Heron J. Mendelian randomisation for mediation analysis: current methods and challenges for implementation. Eur J Epidemiol (2021) 36:465–78. doi: 10.1007/s10654-021-00757-1 PMC8159796 33961203

[17] Birney E. Mendelian randomization. Cold Spring Harb Perspect Med (Cold Spring Harbor, NY: Cold Spring Harbor Laboratory Press) (2022) 12 (4):a041302. doi: 10.1101/cshperspect.a041302 34872952 PMC9121891

[18] Kim SY Park JE Lee YJ Seo H-J Sheen S-S Hahn S. Testing a tool for assessing the risk of bias for nonrandomized studies showed moderate reliability and promising validity. J Clin Epidemiol (2013) 66:408–14. doi: 10.1016/j.jclinepi.2012.09.016 23337781

[19] Kurilshikov A Medina-Gomez C Bacigalupe R Radjabzadeh D Wang J Demirkan A. Large-scale association analyses identify host factors influencing human gut microbiome composition. Nat Genet (2021) 53:156–65. doi: 10.1038/s41588-020-00763-1 PMC8515199 33462485

[20] Day F Karaderi T Jones MR Meun C He C Drong A. Correction: Large-scale genome-wide meta-analysis of polycystic ovary syndrome suggests shared genetic architecture for different diagnosis criteria. PloS Genet (2019) 15:e1008517. doi: 10.1371/journal.pgen.1008517 31805045 PMC6894746

[21] Sanna S van Zuydam NR Mahajan A Kurilshikov A Vich Vila A Võsa U. Causal relationships among the gut microbiome, short-chain fatty acids and metabolic diseases. Nat Genet (2019) 51:600–5. doi: 10.1038/s41588-019-0350-x PMC6441384 30778224

[22] Lv W-Q Lin X Shen H Liu H-M Qiu X Li B-Y. Human gut microbiome impacts skeletal muscle mass via gut microbial synthesis of the short-chain fatty acid butyrate among healthy menopausal women. J Cachexia Sarcopenia Muscle (2021) 12:1860–70. doi: 10.1002/jcsm.12788 PMC8718076 34472211

[23] Jia J Dou P Gao M Kong X Li C Liu Z. Assessment of causal direction between gut microbiota-dependent metabolites and cardiometabolic health: A bidirectional mendelian randomization analysis. Diabetes (2019) 68:1747–55. doi: 10.2337/db19-0153 31167879

[24] Li P Wang H Guo L Gou X Chen G Lin D. Association between gut microbiota and preeclampsia-eclampsia: a two-sample Mendelian randomization study. BMC Med (2022) 20(1):443. doi: 10.1186/s12916-022-02657-x 36380372 PMC9667679

[25] Choi KW Chen C-Y Stein MB Klimentidis YC Wang M-J Koenen KC. Assessment of bidirectional relationships between physical activity and depression among adults. JAMA Psychiatry (2019) 76(4):399–408. doi: 10.1001/jamapsychiatry.2018.4175 30673066 PMC6450288

[26] Hemani G Zheng J Elsworth B Wade KH Haberland V Baird D. The MR-Base platform supports systematic causal inference across the human phenome. eLife (2018) 7:e34408. doi: 10.7554/eLife.34408 29846171 PMC5976434

[27] Emdin CA Khera AV Kathiresan S Mendelian Randomization. JAMA. (2017) 318:1925. doi: 10.1001/jama.2017.17219 29164242

[28] Staiger D Stock JH. Instrumental variables regression with weak instruments. Econometrica (1997) 65:557. doi: 10.2307/2171753

[29] Liu R Zhang C Shi Y Zhang F Li L Wang X. Dysbiosis of gut microbiota associated with clinical parameters in polycystic ovary syndrome. Front Microbiol (2017) 8:324. doi: 10.3389/fmicb.2017.00324 28293234 PMC5328957

[30] Insenser M Murri M Del Campo R Martínez-García MÁ Fernández-Durán E Escobar-Morreale HF. Gut microbiota and the polycystic ovary syndrome: influence of sex, sex hormones, and obesity. J Clin Endocrinol Metab (2018) 103:2552–62. doi: 10.1210/jc.2017-02799 29897462

[31] Zeng B Lai Z Sun L Zhang Z Yang J Li Z. Structural and functional profiles of the gut microbial community in polycystic ovary syndrome with insulin resistance (IR-PCOS): a pilot study. Res Microbiol (2019) 170:43–52. doi: 10.1016/j.resmic.2018.09.002 30292647

[32] Torres PJ Siakowska M Banaszewska B Pawelczyk L Duleba AJ Kelley ST. Gut microbial diversity in women with polycystic ovary syndrome correlates with hyperandrogenism. J Clin Endocrinol Metab (2018) 103:1502–11. doi: 10.1210/jc.2017-02153 PMC6276580 29370410

[33] Zhang J Sun Z Jiang S Bai X Ma C Peng Q. Probiotic Bifidobacterium lactis V9 Regulates the Secretion of Sex Hormones in Polycystic Ovary Syndrome Patients through the Gut-Brain Axis. mSystems (2019) 4(2):e00017–19. doi: 10.1128/msystems.00017-19 PMC6469956 31020040

[34] Zhou L Ni Z Cheng W Yu J Sun S Zhai D. Characteristic gut microbiota and predicted metabolic functions in women with PCOS. Endocrine Connections (2020) 9:63–73. doi: 10.1530/ec-19-0522 31972546 PMC6993273

[35] Jobira B Frank DN Pyle L Silveira LJ Kelsey MM Garcia-Reyes Y. Obese adolescents with PCOS have altered biodiversity and relative abundance in gastrointestinal microbiota. J Clin Endocrinol Metab (2020) 105:e2134–44. doi: 10.1210/clinem/dgz263 PMC7147870 31970418

[36] Liang Y Ming Q Liang J Zhang Y Zhang H Shen T. Gut microbiota dysbiosis in polycystic ovary syndrome: association with obesity — a preliminary report. Can J Physiol Pharmacol (2020) 98:803–9. doi: 10.1139/cjpp-2019-0413 32150694

[37] Chu W Han Q Xu J Wang J Sun Y Li W. Metagenomic analysis identified microbiome alterations and pathological association between intestinal microbiota and polycystic ovary syndrome. Fertil Steril (2020) 113:1286–1298.e4. doi: 10.1016/j.fertnstert.2020.01.027 32482258

[38] Eyupoglu ND Ergunay K Acikgoz A Akyon Y Yilmaz E Yildiz BO. Gut microbiota and oral contraceptive use in overweight and obese patients with polycystic ovary syndrome. J Clin Endocrinol Metab (2020) 105:e4792–800. doi: 10.1210/clinem/dgaa600 32860695

[39] Chen F Chen Z Chen M Chen G Huang Q Yang X. Reduced stress-associated FKBP5 DNA methylation together with gut microbiota dysbiosis is linked with the progression of obese PCOS patients. NPJ Biofilms Microbiomes (2021) 7(1):60. doi: 10.1038/s41522-021-00231-6 34267209 PMC8282850

[40] Lüll K Arffman RK Sola-Leyva A Molina NM Aasmets O Herzig K-H. The gut microbiome in polycystic ovary syndrome and its association with metabolic traits. J Clin Endocrinol Metab (2020) 106:858–71. doi: 10.1210/clinem/dgaa848 33205157

[41] Liang Z Di N Li L Yang D. Gut microbiota alterations reveal potential gut–brain axis changes in polycystic ovary syndrome. J Endocrinological Invest (2021) 44(8):1727–37. doi: 10.1007/s40618-020-01481-5 33387350

[42] Dong S Jiao J Jia S Li G Zhang W Yang K. 16S rDNA full-length assembly sequencing technology analysis of intestinal microbiome in polycystic ovary syndrome. Front Cell Infect Microbiol (2021) 11:634981. doi: 10.3389/fcimb.2021.634981 34041041 PMC8141595

[43] Mammadova G Ozkul C Yilmaz Isikhan S Acikgoz A Yildiz BO. Characterization of gut microbiota in polycystic ovary syndrome: Findings from a lean population. Eur J Clin Invest (2020) 51(4):e13417. doi: 10.1111/eci.13417 32991745

[44] Li G Liu Z Ren F Shi H Zhao Q Song Y. Alterations of gut microbiome and fecal fatty acids in patients with polycystic ovary syndrome in central China. Front Microbiol (2022) 13:911992. doi: 10.3389/fmicb.2022.911992 35847083 PMC9283120

[45] Kåhrström CT Pariente N Weiss U. Intestinal microbiota in health and disease. Nature (2016) 535:47–7. doi: 10.1038/535047a 27383978

[46] Walter K. What is polycystic ovary syndrome? JAMA (2022) 327:294. doi: 10.1001/jama.2021.19776 35040885

[47] Poretsky L. On the paradox of insulin-induced hyperandrogenism in insulin-resistant states. Endocrine Rev (1991) 12:3–13. doi: 10.1210/edrv-12-1-3 2026121

[48] Guo Y Qi Y Yang X Zhao L Wen S Liu Y. Association between polycystic ovary syndrome and gut microbiota. PloS One (2016) 11:e0153196. doi: 10.1371/journal.pone.0153196 27093642 PMC4836746

[49] Kelley ST Skarra DV Rivera AJ Thackray VG. The gut microbiome is altered in a letrozole-induced mouse model of polycystic ovary syndrome. PloS One (2016) 11:e0146509. doi: 10.1371/journal.pone.0146509 26731268 PMC4701222

[50] Torres PJ Ho BS Arroyo P Sau L Chen A Kelley ST. Exposure to a healthy gut microbiome protects against reproductive and metabolic dysregulation in a PCOS mouse model. Endocrinology (2019) 160:1193–204. doi: 10.1210/en.2019-00050 PMC6482036 30924862

[51] He Y Wu W Zheng H-M Li P McDonald D Sheng H-F. Regional variation limits applications of healthy gut microbiome reference ranges and disease models. Nat Med (2018) 24:1532–5. doi: 10.1038/s41591-018-0164-x 30150716

[52] Davey Smith G Ebrahim S. “Mendelian randomization”: can genetic epidemiology contribute to understanding environmental determinants of disease? Int J Epidemiol (2003) 32:1–22. doi: 10.1093/ije/dyg070 12689998

[53] Ference BA. How to use Mendelian randomization to anticipate the results of randomized trials. Eur Heart J (2017) 39:360–2. doi: 10.1093/eurheartj/ehx462 29020392

[54] Seo B Yoo JE Lee YM Ko G. Sellimonas intestinalis gen. nov., sp. nov., isolated from human faeces. Int J Syst Evol Microbiol (2016) 66:951–6. doi: 10.1099/ijsem.0.000817 26637816

[55] Muñoz M Guerrero-Araya E Cortés-Tapia C Plaza-Garrido Á Lawley TD Paredes-Sabja D. Comprehensive genome analyses of Sellimonas intestinalis, a potential biomarker of homeostasis gut recovery. Microbial Genomics (2020) 6(12):mgen000476. doi: 10.1099/mgen.0.000476 33206037 PMC8116674

[56] Liang X Wang R Luo H Liao Y Chen X Xiao X. The interplay between the gut microbiota and metabolism during the third trimester of pregnancy. Front Microbiol (2022) 13:1059227. doi: 10.3389/fmicb.2022.1059227 36569048 PMC9768424

[57] Yang R Shan S Shi J Li H An N Li S. Coprococcus eutactus, a Potent Probiotic, Alleviates Colitis via Acetate-Mediated IgA Response and Microbiota Restoration. J Agric Food Chem (2023) 7:3273–84. doi: 10.1021/acs.jafc.2c06697 36786768

[58] Suzuki TA Fitzstevens JL Schmidt VT Enav H Huus KE Mbong Ngwese M. Codiversification of gut microbiota with humans. Science (2022) 377:1328–32. doi: 10.1126/science.abm7759 PMC10777373 36108023

[59] Verheggen RJHM Konstanti P Smidt H Hermus ARMM Thijssen DHJ Hopman MTE. Eight-week exercise training in humans with obesity: Marked improvements in insulin sensitivity and modest changes in gut microbiome. Obesity (2021) 29:1615–24. doi: 10.1002/oby.23252 PMC9291576 34467673

[60] Akcalı A Bostanci N Özçaka Ö Öztürk-Ceyhan B Gümüş P Buduneli N. Association between polycystic ovary syndrome, oral microbiota and systemic antibody responses. PloS One (2014) 9:e108074. doi: 10.1371/journal.pone.0108074 25232962 PMC4169459

[61] Lu C Wang H Yang J Zhang X Chen Y Feng R. Changes in vaginal microbiome diversity in women with polycystic ovary syndrome. Front Cell Infect Microbiol (2021) 11:755741. doi: 10.3389/fcimb.2021.755741 34804995 PMC8596286

[62] Guo X Okpara ES Hu W Yan C Wang Y Liang Q. Interactive relationships between intestinal flora and bile acids. Int J Mol Sci (2022) 23:8343. doi: 10.3390/ijms23158343 35955473 PMC9368770

[63] González F. Inflammation in Polycystic Ovary Syndrome: Underpinning of insulin resistance and ovarian dysfunction. Steroids (2012) 77:300–5. doi: 10.1016/j.steroids.2011.12.003 PMC3309040 22178787

[64] Zhao X Jiang Y Xi H Chen L Feng X. Exploration of the relationship between gut microbiota and polycystic ovary syndrome (PCOS): a review. Geburtshilfe und Frauenheilkunde (2020) 80:161–71. doi: 10.1055/a-1081-2036 PMC7035130 32109968

[65] Assimakopoulos SF Tsamandas AC Louvros E Vagianos CE Nikolopoulou VN Thomopoulos KC. Intestinal epithelial cell proliferation, apoptosis and expression of tight junction proteins in patients with obstructive jaundice. Eur J Clin Invest (2010) 41:117–25. doi: 10.1111/j.1365-2362.2010.02379.x 20840373

[66] Turnbaugh PJ Ley RE Mahowald MA Magrini V Mardis ER Gordon JI. An obesity-associated gut microbiome with increased capacity for energy harvest. Nature (2006) 444:1027–31. doi: 10.1038/nature05414 17183312

[67] Walters WA Xu Z Knight R. Meta-analyses of human gut microbes associated with obesity and IBD. FEBS Lett (2014) 588:4223–33. doi: 10.1016/j.febslet.2014.09.039 PMC5050012 25307765

[68] Tilman D. Diversity and productivity in a long-term grassland experiment. Science (2001) 294:843–5. doi: 10.1126/science.1060391 11679667

[69] Bickerton AST Clark N Meeking D Shaw KM Crook M Lumb P. Cardiovascular risk in women with polycystic ovarian syndrome (PCOS). J Clin Pathol (2005) 58:151–4. doi: 10.1136/jcp.2003.015271 PMC1770573 15677534

[70] Pedersen HK Gudmundsdottir V Nielsen HB Hyotylainen T Nielsen T Jensen BAH. Human gut microbes impact host serum metabolome and insulin sensitivity. Nature (2016) 535:376–81. doi: 10.1038/nature18646 27409811

[71] Cani PD Amar J Iglesias MA Poggi M Knauf C Bastelica D. Metabolic endotoxemia initiates obesity and insulin resistance. Diabetes (2007) 56:1761–72. doi: 10.2337/db06-1491 17456850

[72] Moreira S Soares E Tomaz G Maranhão T Azevedo G. [Polycystic ovary syndrome: a psychosocial approach]. Acta Med Portuguesa (2010) 23:237–42. doi: 10.20344/amp.611 20470471

[73] Wang MH Cordell HJ Van Steen K. Statistical methods for genome-wide association studies. Semin Cancer Biol (2019) 55:53–60. doi: 10.1016/j.semcancer.2018.04.008 29727703

[74] Davey Smith G Hemani G. Mendelian randomization: genetic anchors for causal inference in epidemiological studies. Hum Mol Genet (2014) 23:R89–98. doi: 10.1093/hmg/ddu328 PMC4170722 25064373

[75] Curtin F Schulz P. Multiple correlations and bonferroni’s correction. Biol Psychiatry (1998) 44:775–7. doi: 10.1016/s0006-3223(98)00043-2 9798082

[76] Sham PC Purcell SM. Statistical power and significance testing in large-scale genetic studies. Nat Rev Genet (2014) 15 (5):335–46. doi: 10.1038/nrg3706 24739678

[77] Yarmolinsky J Wade KH Richmond RC Langdon RJ Bull CJ Tilling KM. Causal inference in cancer epidemiology: what is the role of mendelian randomization? Cancer Epidemiol Biomarkers Prev (2018) 27 (9):995–1010. doi: 10.1158/1055-9965.EPI-17-1177 PMC6522350 29941659

[78] Armstrong RA. When to use the Bonferroni correction. Ophthalmic Physiol Opt (2014) 34 (5):502–8. doi: 10.1111/opo.12131 24697967

